# Genetic and clinical characterization of *BRCA*-associated hereditary breast and ovarian cancer in Navarra (Spain)

**DOI:** 10.1186/s12885-019-6277-x

**Published:** 2019-11-27

**Authors:** Ainara Ruiz de Sabando, Edurne Urrutia Lafuente, Fermín García-Amigot, Angel Alonso Sánchez, Lourdes Morales Garofalo, Sira Moreno, Eva Ardanaz, Maria A. Ramos-Arroyo

**Affiliations:** 1 Department of Medical Genetics, Complejo Hospitalario de Navarra (CHN), Pamplona, Spain; 2 Navarrabiomed, Pamplona, Spain; 3 IdiSNA, Navarra Institute for Health Research, Pamplona, Spain; 4 Navarra Public Health Institute, Pamplona, Spain; 5 CIBER Epidemiology and Public Health CIBERESP, Madrid, Spain

**Keywords:** Hereditary breast and ovarian cancer (HBOC), *BRCA1/2*, Recurrent mutations, Demographics, Sporadic breast and ovarian cancer, Laterality and stage of tumors, Overall survival, Navarra

## Abstract

**Background:**

Genetic testing for *BRCA1/2* genes is widely used as a strategy to reduce incidence and morbidity of hereditary breast and ovarian cancer (HBOC). The purpose of this study is to analyse the demographic and molecular characteristics of *BRCA* germline mutations in Navarra, Spain, and to investigate the clinical profile of hereditary and sporadic breast cancer (BC) and ovarian cancer (OC) in the Community.

**Methods:**

The study includes 1246 individuals assessed for *BRCA1/2* genetic testing in Navarra, during 2000–2016, and a cohort of BC (*n* = 4384) and OC (*n* = 561) from the population-based Navarra Cancer Registry. Distribution and molecular characteristics of *BRCA1/2* mutations, as well as, comparative analysis of the clinical course, pathologic features and overall survival (OS) of patients in different risk groups were investigated.

**Results:**

*BRCA* mutation detection rate was 16%, with higher proportion (63%) of *BRCA2* families. Nineteen per cent of mutations were recurrent, one of which, *BRCA2* c.6024dupG, showed high association to OC. *BRCA* carriers had double risk (95% CI = 1.04–4.33) of developing multiple malignancies than low risk families and were diagnosed at a much earlier age (16.6 and 11.7 years difference for BC and OC, respectively) when compared to the general population. For BC, *BRCA* carriers showed a more advanced histological stage, higher risk of bilateral neoplasms (OR = 4.3; 95% CI = 1.3–11.4, for *BRCA2* carriers) and worse OS rate at 5-, 10- and 15- years, than women with sporadic tumors. For OC, over 70% of patients of all risk groups showed advanced stages at diagnosis, with the highest among *BRCA1* carriers (91%). Furthermore, they also had higher probability of developing ovarian bilateral tumors (OR = 7.8, 95% CI = 1.7–55.7, for *BRCA1* carriers) than the general population. Five-year OS rate was worse among women with sporadic OC than *BRCA* carriers, but it levelled out over the 15-year period.

**Conclusions:**

In addition to national similarities in the HBOC-*BRCA1/2* associated mutational spectrum, we identified a recurrent *BRCA2* pathogenic variant (c.6024dupG), highly associated to OC in Navarra. Carriers of *BRCA1/2* mutations showed a more severe BC and OC phenotype and had a worse overall prognosis when compared to a large cohort of women with sporadic counterpart tumors.

## Background

About 32,800 breast (BC) and 3300 ovarian cancers (OC) are diagnosed every year in Spain, accounting for one third of the cancers among women [1–3]. Although survival has substantially improved over the past few years due to screening and the improvement of treatments, BC is the leading cause of cancer death among Spanish women, with about 6500 deaths in 2017, and OC is the fifth, with 2000 [4]. Between 5 and 10% [5] of all BC and 13–15% of OC [6, 7] are hereditary and approximately 25% are associated with the Hereditary breast/ovarian cancer (HBOC) syndrome, caused by abnormalities in the DNA repair genes *BRCA1* and *BRCA2* [8]. Inherited mutations in the *BRCA1* and *BRCA2* (*BRCA1/*2) cancer susceptibility genes convey high lifetime risks of BC and OC, in the range of 40–66% and 13–46%, respectively [9, 10], as well as, increased susceptibility for other malignancies, such as prostate and pancreatic cancers [11]. On the other hand, it has been hypothesized that *BRCA*-associated BC has a different prognosis as compared to the sporadic counterpart [12]. However, clinical findings regarding the prognostic role of *BRCA* mutational status are still controversial.

Genetic testing for *BRCA1/2* mutations allows us to stratify families and individuals by their risk or predisposition to developing cancer, so that, preventive measurements can be offered to decrease cancer mortality and morbidity. Characterization of the most prevalent *BRCA1* and *BRCA2* mutations and their geographical distribution can be useful in designing efficient mutational screening in a given population. Additionally, it is important to evaluate genetic testing and preventive programs for HBOC, carrying out intermediate processes of data analysis and comparison of outcome indicators.

Previous studies have analysed the mutation spectrum of *BRCA1/2* in different regions of Spain [13–19]. Navarra, with about 650,000 people in 2018, has historically been in exchange with the neighbouring Basque territories in the north and Aragon and Castile in the southeast; additionally, in the last decades, it has experienced migration from other Spanish communities and countries. The aim of the present study is two-fold: a) to describe the molecular and demographic characteristics of families with HBOC syndrome in Navarra, Spain, b) to define the associated clinical phenotype and overall survival (OS) of individuals with BC and OC in different hereditary cancer risk families and the general population, in an attempt to provide indicators that may allow us to evaluate and improve the HBOC testing program in our community.

## Methods

### Hereditary breast/ovarian cancer study population

From 2000 to 2016, a total of 1246 individuals of Navarra, corresponding to 751 families with BC and/or OC, were evaluated at the Hereditary Cancer Clinic of the Department of Medical Genetics of the Complejo Hospitalario de Navarra (CHN), the reference public center for the HBOC program in the Autonomous Community. Since 2000, families with suspected familial/hereditary cancers are referred by medical specialists to the CHN for evaluation, counselling and genetic testing, if considered appropriate. Relevant information including personal and familial history, age of cancer diagnosis, tumor type and geographic family origin, is routinely obtained. Families are classified as high-risk families, if they meet the criteria established by the Breast Cancer Committee of the CHN (Additional file 1: Figure S1), or as low-risk families, if not. High-risk families are informed about their hereditary cancer risk, advised according to their personal and familial circumstances and offered genetic analysis of *BRCA1* and *BRCA2* genes. Additionally, members of *BRCA1/2* positive families that request genetic testing are studied and followed according to their individual risk status. All tested individuals provide a signed informed consent, following the appropriate genetic counselling. For this study, a disaggregated identification number was assigned to all participants to guarantee confidentiality and data protection. The study was approved by the Research Ethical Committee of Navarra.

### Origin of families

Geographical origin of families was assessed by enquiring about the place of birth of parents and grandparents of index individuals. Positive *BRCA1/2* families were classified in one of the following geographic groups, based on the place of origin of their ancestors: a) Navarra, when the transmitting parent and/or grandparents were born in the Community of Navarra, b) neighbouring communities, when the transmitting parent and/or grandparents were from the surrounding communities of Navarra (Basque Country, Rioja, Soria, Zaragoza and Huesca), c) other Spanish communities, and d) other countries. The results were represented using the Geographic Information System QGIS version 3.0 Girona.

### Mutation analysis of *BRCA1* and *BRCA2*

Genomic DNA was extracted from peripheral blood of index cases of high-risk families. Sanger sequencing of *BRCA1* and *BRCA2* genes was performed using BigDye® Terminator kits and read through 3500 Genetic Analyzer (Applied BioSystems). Mutational analysis was performed with SeqScape Software v3.0 (Thermo Fisher Scientific). All index patients were additionally tested for the presence of Large Genomic Rearrangements in *BRCA1* and *BRCA2* genes by Multiplex ligation-dependent probe amplification (MLPA). Specific probes for each exon of *BRCA1* (SALSA MLPA P002 and P087 *BRCA1* probemix, MRC-Holland) and *BRCA2* genes (SALSA MLPA P045 *BRCA2*/*CHEK2* probemix, MRC-Holland) were used. The fragments were measured by capillary electrophoresis using the 3500 Genetic Analyzer (Applied Biosystems) and analyzed with Coffalyzer (MRC-Holland).

### Variant nomenclature & classification

GenBank reference sequences of NM_007294.3 and NM_000059.3 were used for *BRCA1 and BRCA2* analysis*,* respectively. Mutation nomenclature was described according to Human Genome Variation Society [20]. Pathogenicity of the mutations was assessed using the following databases: the Breast Cancer Information Core [21], the Universal Mutation Database [22] for *BRCA1* and *BRCA2*, and ClinVar at NCBI [23]. For novel mutations the standards and guidelines of the American College of Medical Genetics and Genomics were followed to determine the possible disease-association [24]. In silico prediction was evaluated considering the impact of the amino acid changes as well as their conservation across species. These analyses were performed with the following bioinformatic tools: PROtein Variation Effect Analyzer (PROVEAN) [25], Polymorphism Phenotyping-2 (PolyPhen-2) [26].

### Clinical and pathological features of *BRCA* and non-*BRCA* related breast and ovarian cancer. Navarra Cancer Registry

Individuals of Navarra are registered for health care and administrative purposes by a unique personal identification number in regional medical registries. This number was used to cross-link the information between the HBOC study and the Cancer Registry of Navarra, a regional registry that, since 1973, records all tumor malignancies of Navarra residents. We used this Registry as a reliable source of clinical information on a total of 596 participants of the HBOC study that had developed, at least, one infiltrating cancer, before January 2013. For comparison with tumors of sporadic origin, we also analysed a cohort sample of 4384 cases with BC and 561 with OC diagnosed in the general population from 2000 to 2012. The following variables were obtained: gender, number of infiltrating tumors per patient, age at diagnosis of each cancer, age at death, laterality, site, histology and stage of neoplasms. Site and histology were defined according to the International Classification of Diseases for Oncology (ICD-O) [27]. The extent of the disease was coded according to the Condensed TNM of the European Network of Cancer Registries [28].

### Statistics

Statistical analysis was performed using IBM SPSS Statistics for Windows, Version 20.0 and OpenEpi [29]. Socio-demographic and clinical variables were summarized using descriptive statistics, such as mean and standard deviation (SD) and frequencies and percentages. The associated risk of developing any tumor, of having bilateral tumor, and of having regional/advanced stage by HBOC group, compared with that of the general population (Cancer Registry), was estimated using odds ratios (OR), with 95% confidence intervals. The association between selected clinical–pathological data and specific groups of HBOC, all categorical variables, was determined by using Chi-Square or Fisher’s exact test, and Student’s T test, as appropriate. Student’s T test was used to compare independent continuous variables. Kaplan-Meier survival curves (proportion surviving) with 5–10- and 15-year survival were plotted, and log-rank test was used to compare different groups. Cox (hazard ratios, HR) regression was also performed, adjusting for age at diagnosis. All tests were two-sided and *p*-value less than 0.05 was considered to be statistically significant.

## Results

### Hereditary breast/ovarian cancer study (2000–2016)

**Fig. 1:**
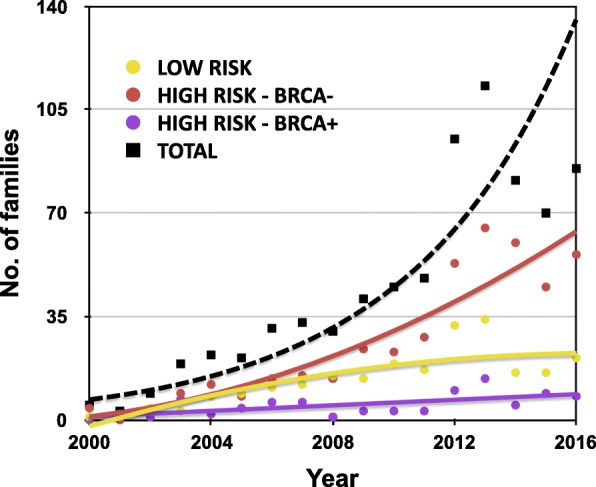
Number of families included in the hereditary breast/ovarian cancer study (2000–2016)

Among the 751 evaluated families, residents of Navarra, with clinical and family history that suggested HBOC, 233 were classified as low-risk families, with no further analysis, and 518 others as high-risk families, for which mutational analysis was performed in the index affected member (Table 1). The number of cases included in the study is represented in Fig. 1, showing a remarkable increase of *BRCA1/2* testing and diagnosis over time.

**Table 1:** Total number of subjects and families included in the hereditary breast/ovarian cancer (HBOC) study

	Total	HBOC Study			
		High risk *BRCA1*+ *n* (%)	High risk *BRCA2*+ *n* (%)	High risk *BRCA*- *n* (%)	Low risk *n* (%)
No. Families	751	31 (4.1%)	53 (7.1%)	434 (57.8%)	233 (31%)
No. Individuals	1246	94 (7.5%)	135 (10.8%)	648 (52%)	369 (29.6%)

**Fig. 2:**
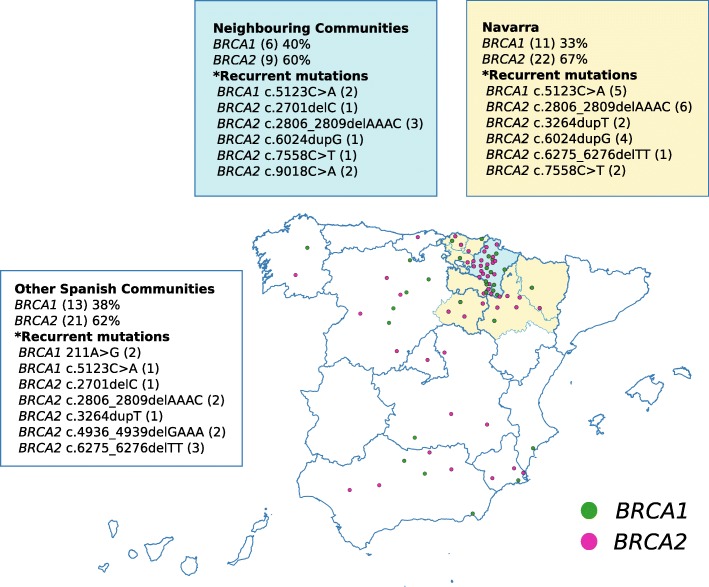
Geographical origin of the hereditary breast/ovarian cancer families in Navarra. (Map created with QGIS 3.0)

From the high-risk group, 84 unrelated families (16.2%), 228 individuals, were found carrying pathogenic mutations: 31 (37%) in *BRCA1* and 53 (63%) in *BRCA2*. Of them, 39% of the transmitting parents or grandparents were originally from Navarra (33 families), 18% came from the neighbouring communities (15 families) and the remaining families (42%) had ancestors from other communities (34 families) or countries (2 families) (Fig. 2). Frequency of mutations in *BRCA2* gene was higher than in *BRCA1* in all geographic groups, and more so in families of local ancestors, in which two thirds (67%) of them were *BRCA2* positive.

*BRCA1* families (*n* = 31) carried a total of 23 different mutations (Additional file 2: Table S1); two were recurrent mutations and 21 were unique mutations. The most frequent mutation was c.5123C > A, present in eight apparently unrelated families, followed by c.211A > G, identified in two families, representing a 26, and 7%, respectively, of the total families with *BRCA1* mutations.

Among *BRCA2* positive families (*n* = 53), 29 different mutations were observed (Additional file 3: Table S2). Eight were recurrent mutations and 21 unique mutations. Variant c.2806_2809delAAAC was highly represented (11 unrelated families). The five most frequent pathogenic variants accounted for 50% of *BRCA2* mutations.

Four novel, unreported, pathogenic mutations, responsible for early-onset BC and OC were identified.

#### *BRCA1* c.4343delG (exon 13)

This deletion, located in exon 13, results in a frame shift variant that originates a stop codon in position 1455. The index patient in this family was a male, diagnosed of PC at age 67 with family history of BC and OC on the mother’s side. BCs were diagnosed at ages 90 (mother) and 55 (maternal aunt) and OC at age 42 (maternal aunt).

#### *BRCA1* exon 5–7 duplication

This variant was found in a 54 year old woman diagnosed with invasive ductal carcinoma. Her mother died of BC at 46. Two additional family members (a maternal cousin and her daughter), carriers of the same mutation, suffered from BC at ages 33 and 34, respectively.

#### *BRCA2* c.4132_4133delAC (exon 11)

This alteration triggers a frame shift variant resulting in a stop codon in position 1380. The index patient, who inherited the mutation from her father, was diagnosed with invasive ductal carcinoma at 40 years old and a few months after she also developed cervix cancer. Two of her paternal aunts died of BC.

#### *BRCA2* c.5216_5218insAAA (exon 11)

This insertion originates a nonsense mutation with a stop codon in position 1739. The index patient was a woman that developed BC at 28 years old, whose mother, carrier of the mutation, had OC at the age of 60.

### Clinical data from the Navarra Cancer Registry

Of all the 1246 individuals in the HBOC study, 593 were included in the Navarra Cancer Registry with a total of 693 tumor entries. Twenty four (4%) patients were males and 9 (37.5%) of them carried a *BRCA* deleterious mutation.

The frequency of multiple tumors was significantly higher among *BRCA1/2* carriers (21.6%) than in individuals of the low-risk families (11.5%), with an OR of 2.11 (95% CI = 1.04 to 4.33, *p* = 0.038) (Table 2). The difference did not reach statistical significance when compared with the high risk *BRCA*-negative group (HR-*BRCA*-negative). *BRCA1* and *BRCA2* mutations were associated with a different range of tumors. Cancer affecting breast, ovary, skin (SC), endometrium (EC), pancreas (PC) and prostate (PrC) accounted for 91.2% of all tumors registered in *BRCA1/2* patients and 93.7% among HR-*BRCA*-negative and low risk cases. *BRCA1* carriers had a higher frequency of OC (24.1%), SC (11.1%) and PC (3.7%) than individuals in the other three groups (9.3, 5.3 and 0%, respectively). BC (70.7%) and PrC (2.7%), however, were overrepresented in *BRCA2* carriers compared to *BRCA1*-positives (55.6 and 0%, respectively).

**Table 2:** Number and type of malignancies among individuals of the hereditary breast/ovarian cancer (HBOC) study

		Total	HBOC study			
			High risk BRCA1+ *n* (%)	High risk BRCA2+ *n* (%)	High risk BRCA- *n* (%)	Low risk *n* (%)
Navarra Cancer Registry	Patients with one or more tumors					
	Single tumor	506 (85.3%)	33 (78.6%)	47 (78.3%)	303 (86.1%)	123 (88.5%)
	Multiple tumors (a)	87 (14.7%)	9 (21.4%)	13 (21.7%)	49 (13.9%)	16 (11.5%)
	Total	593	42	60	352	139
	(a) *BRCA1/2* vs Low Risk: OR = 2.11 95%CI = 1.04 to 4.33, *p*-value = 0.038					
	Tumors by affected tissue					
	BREAST	531 (76.6%)	30 (55.6%)	53 (70.7%)	327 (80.5%)	120 (76.4%)
	OVARY	55 (7.9%)	13 (24.1%)	7 (9.3%)	27 (6.6%)	8 (5.1%)
	SKIN	46 (6.6%)	6 (11.1%)	4 (5.3%)	24 (5.9%)	12 (7.6%)
	ENDOMETRIUM	13 (1.9%)	1 (1.8%)	2 (2.7%)	7 (1.7%)	3 (1.9%)
	PANCREAS	5 (0.7%)	2 (3.7%)	0	0	3 (1.9%)
	COLON	5 (0.7%)	0	1 (1.3%)	4 (1%)	0
	PROSTATE GLAND	3 (0.4%)	0	2 (2.7%)	0	1 (0.6%)
	OTHERS	35 (5.1%)	2 (3.7%)	6 (8%)	17 (4.2%)	10 (6.4%)
	TOTAL	693	54	75	406	157

Among men, there were 3 *BRCA1* mutation carriers who developed PC (1) and SC (2), and 6 *BRCA2* positive cases, presenting with a wide variety of tumors: breast (4), prostate gland (2), lung (1), colon (1), fossa piriform (1) and thyroid gland (1). Three of the 9 *BRCA1/2* male mutation carriers, developed multiple tumors, all associated with *BRCA2*.

To further study severity of the disease we analysed laterality, stage of the tumor, age at diagnosis and overall survival (OS) in patients of the HBOC study in comparison with a cohort sample of BC (*n* = 4384) and OC (*n* = 561) in the general population. As shown in Table 3, all four risk groups of the HBOC study showed a higher incidence of bilateral BC compared to the general population, although only in the group of *BRCA2* positives the difference reached statistical significance (7.6% vs 1.8%; *p* = 0.021, OR = 4.3; 95% CI = 1.3 to 11.4). For OC, *BRCA1* carriers had a much higher frequency of bilateral tumors (77.8%) than individuals in the *BRCA2* positive (33.3%), HR-*BRCA* negative (30.8%), low-risk HBOC (0%) and the general population (30.9%; *p* = 0.007, OR = 7.8; 95% CI = 1.7 to 55.7).

**Table 3:** Clinical characteristics of breast and ovarian cancer cases included in the study

	Hereditary breast/ovarian cancer study				Cancer Registry	*P*-value
	High risk *BRCA1*+ *n* (%)	High risk *BRCA2*+ *n* (%)	High risk *BRCA*- *n* (%)	Low risk *n* (%)		
Breast cancer						
Laterality						
Right	11 (37.9%)	22 (41.5%)	153 (48.1%)	51 (44%)	2003 (47.4%)	
Left	17 (58.6%)	27 (50.9%)	155 (48.7%)	61 (52.6%)	2142 (50.7%)	
Bilateral	1 (3.5%)	4 (7.6%)	10 (3.2%)	4 (3.5%)	78 (1.8%)	0.021 (a)
Total	29	53	318	116	4223	
(a) *BRCA2+* vs Cancer Registry: OR = 4.3; 95% CI = 1.3 to 11.4						
Stage						
Localized	10 (40%)	24 (47.1%)	154 (57.2%)	56 (59.6%)	2396 (57%)	
Regional	13 (52%)	24 (47.1%)	105 (39%)	36 (38.3%)	1618 (38.5%)	
Advanced	2 (8%)	3 (5.9%)	10 (3.7%)	2 (2.1%)	189 (4.5%)	0.043 (b)
Total	25	51	269	94	4203	
(b) *BRCA1/2* vs Cancer Registry						
Age at diagnosis in years						
Mean (standard deviation)	44.3 (8.98)	44.12 (10.17)	45.72 (10.34)	50.14 (11.44)	60.79 (15.47)	
Number	30	53	327	120	4384	
p-value	< 0.001	< 0.001	< 0.001	< 0.001	Reference	
Ovarian cancer						
Laterality						
Right	1 (11.1%)	0	6 (46.2%)	3 (60%)	100 (37.2%)	
Left	1 (11.1%)	2 (66.7%)	3 (23.1%)	2 (40%)	86 (32%)	
Bilateral	7 (77.8%)	1 (33.3%)	4 (30.8%)	0	83 (30.9%)	0.007 (c)
Total	9	3	13	5	269	
(c) *BRCA1+* vs Cancer Registry: OR = 7.8; 95% CI = 1.7 to 55.7						
Stage						
Localized	1 (9.1%)	1 (25%)	6 (25%)	2 (28.6%)	28 (23%)	
Advanced	10 (90.9%)	3 (75%)	18 (75%)	5 (71.4%)	94 (77%)	0.43 (d)
Total	11	4	24	7	122	
(d) *BRCA1/2* vs Cancer Registry						
Age at diagnosis in years						
Mean (standard deviation)	54.12 (12.03)	53.33 (14.68)	53.29 (10.86)	54.55 (13.08)	65.63 (15.43)	
Number	13	7	27	8	561	
*p*-value	0.007	0.078	< 0.001	0.057	Reference	

The stage at diagnose of BC tumors was significantly more advanced (*p* = 0.043) in *BRCA1*/*2* patients than in sporadic tumors. Over 55% of tumors were at regional/advanced stage among *BRCA* carriers compared to 40.4 and 43% in the low risk HBOC and the general population, respectively. The same trend was observed for OC among *BRCA1* carriers, although numbers were too small to reach statistical significance.

**Fig. 3:**
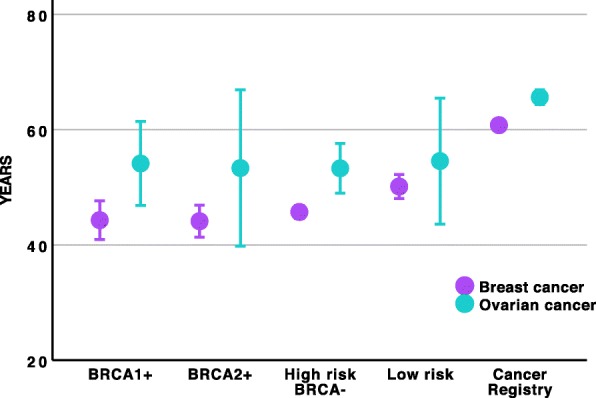
Mean age at diagnosis of breast and ovarian cancer cases included in the study

Mean age at diagnose of BC and OC was lower in all four groups of the HBOC study than in the general population (Table 3, Fig. 3). *BRCA1/2* carriers were diagnosed of BC 16.6 years earlier (44.2 vs 60.8 years), while for OC the time difference was 11.7 years (53.9 vs 65.6).

**Fig. 4:**
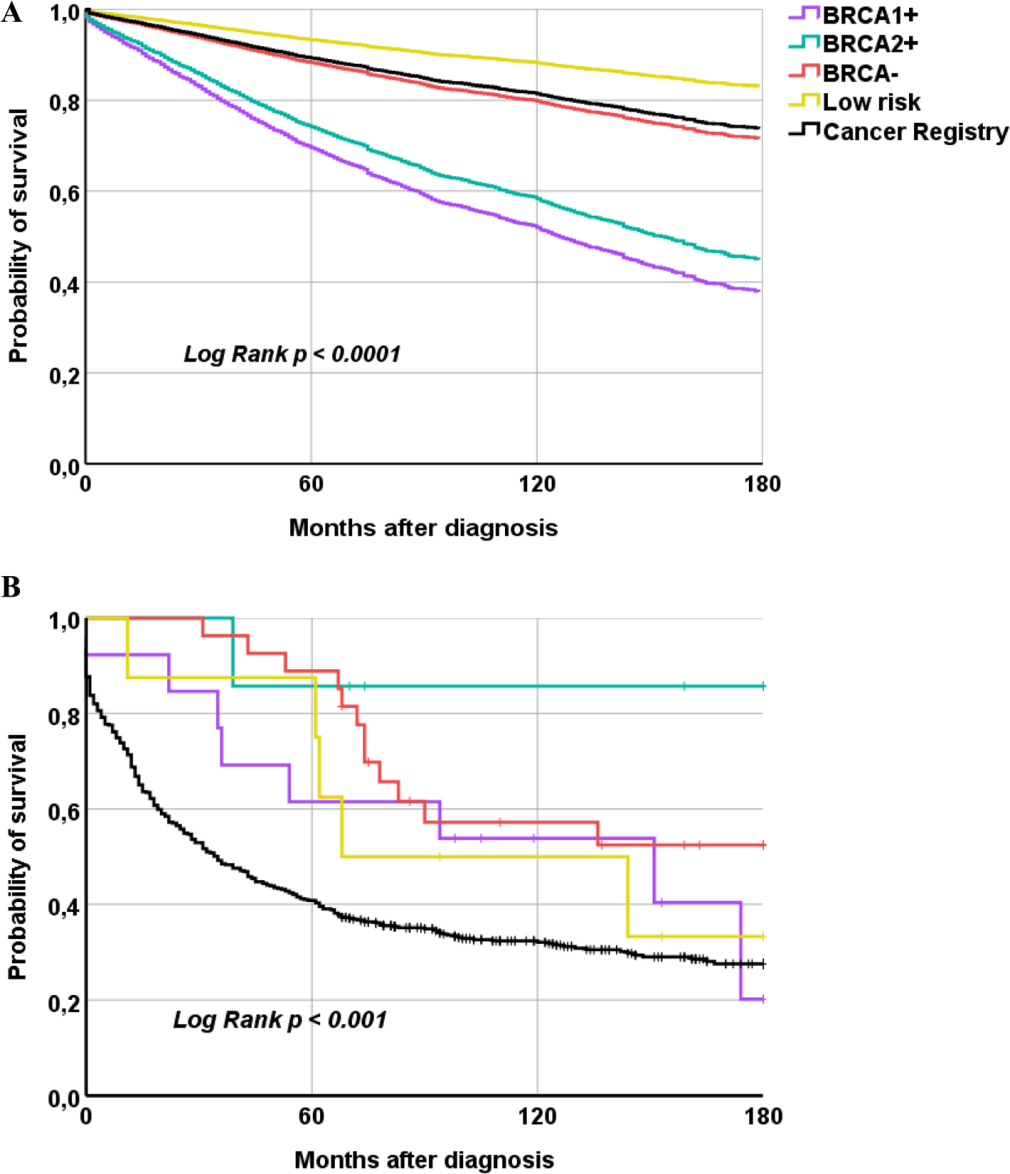
Overall survival curves for breast and ovarian cancer. **a** Cox regression adjusted by age at diagnosis for breast cancer; **b** Kaplan-Meier curves for ovarian cancer

Survival over the course of 15 years was also studied. For BC, 5–10- and 15-year OS did not show significant differences among risk groups (Table 4). More than 64% of *BRCA* carriers lived 15 or more years after diagnosis, a percentage similar to that observed in sporadic BC. However, mean age at death differed between groups. *BRCA1/2* mutation carriers died 20 years earlier than BC patients from the general population, indicating that age at diagnosis could be a co-variant of prognosis (HR = 1.08; 95% CI =1.07 to 1.08; *p* < 0.0001). Further analysis, adjusting by age at diagnosis, showed that *BRCA1* and *BRCA2* carriers had a significant worse prognosis than individuals of the other groups (Fig. 4a), with HR of 3.2 (95% CI = 1.5 to 6.7; *p* = 0.002) and 2.6 (95%CI = 1.5 to 4.7; *p* = 0.001) respectively, when compared with BC of sporadic origin.

**Table 4:** Overall survival rates for breast and ovarian cancer

	Number	Mean age at death	Overall Survival			Log Rank test	Age adjusted	
			5-year	10-year	15-year		Hazard Ratio (95% CI)	*P*-value
Breast Cancer								
*BRCA1*+	30	58.79 ± 13.96	0.863	0.823	0.640	*p* < 0.001	3.19 (1.51, 6.74)	0.002
*BRCA2*+	53	53.4 ± 11.06	0.882	0.786	0.703		2.63 (1.48, 4.67)	0.001
2High risk *BRCA*-	327	50.73 ± 11.87	0.948	0.899	0.840		1.1 (0.79, 1.54)	0.578
Low risk	120	55.41 ± 11.49	0.967	0.936	0.874		0.61 (0.33, 1.14)	0.122
Cancer Registry	4384	77.12 ± 15.83	0.818	0.724	0.651		Reference	
Ovarian Cancer								
*BRCA1*+	13	62.68 ± 13.43	0.615	0.538	0.202	*p* < 0.001	–	
*BRCA2*+	7	53.93	0.857	0.857	0.857			
High risk *BRCA*-	27	64.1 ± 10.05	0.889	0.572	0.525			
Low risk	8	64.46 ± 12.27	0.875	0.500	0.333			
Cancer Registry	561	72.85 ± 12.37	0.408	0.321	0.276

For OC, the general population showed worst 5-year OS rate (41%), becoming, however, similar in all risk groups at 15 years after diagnosis, with OS rates of 20% in *BRCA1* mutation carriers and 28% in the general population (Table 4, Fig. 4b). Mean age at death differed notoriously, but sample size was not sufficient to use the modelling approach as for BC analysis. Nevertheless, when we analysed OS only in patients diagnosed before the age of 65 years, no significant differences were observed among risk groups (data not shown).

## Discussion

We present a demographic, clinical and molecular study of families with HBOC in Navarra (Spain), through the analysis of retrospective data collected by the Department of Medical of the CHN, and the Cancer Registry for the Autonomous Community.

Mutations in *BRCA1* and *BRCA2* genes were identified in 16% of families (26% of individuals) included in the high-risk HBOC sample. This rate is comparable with other studies [14, 18, 19] and similarly shows a decreasing trend over time, moving from 30% in 2000–2005, to 17% during (2006–2010) and 14% from 2011 to 2016. This is, most likely, due to the referral of the most severely affected families in the first years of cancer genetic services, and the use of less strict inclusion criteria in the later period, given the decrease in cost of the molecular analysis. It is noteworthy to highlight, however, the remarkable growth in the number of families and individuals assessed along the study period. Two thirds of the high-risk families were diagnosed in the last 6 years of the study, accounting for more than half of the families with pathogenic variants in *BRCA1* and *BRCA2.*

Navarra is a small community in the North of Spain that has experienced important demographic changes since the 1950 decade, due to migration from other Spanish communities or countries. This mixed population could explain, at least in part, the similarities in *BRCA1/2* mutation distribution and spectrum with other areas of Spain [17, 19]. As previously reported national wide, with the exception of Galicia and Asturias [13, 14], deleterious mutations in *BRCA2* were more frequent than in *BRCA1*, particularly among families of local ancestors in which 2/3 of *BRCA* associated families carried a *BRCA2* deleterious variant. Nineteen per cent (10/52) of pathogenic variants were recurrent and were responsible of 50% of HBOC families (Additional files 2 and 3: Tables S1 and S2). Many common mutations in Navarra are also frequent in other Spanish populations [14–18]. More so, many of the recurrent mutations in Navarra coincide with the most frequent *BRCA1/2* variants in Spain, as published in a recent international study [30].

In addition to these national similarities, *BRCA1/2* mutations in Navarra present some unique features. Recurrent deleterious variants show geographic clusters in more isolated regions. Our most frequent mutation, *BRCA2* c.2806_2809delAAAC, is mainly distributed in the north-western mountainous lands, while the also frequent *BRCA2* c.6024dupG is present in the south-western riverbanks. Interestingly, *BRCA2* c.6024dupG and c.7558C > T, are two recurrent mutations not reported previously in Spain. *BRCA2* c.7558C > T, also found (non-recurrently) in Sweden and Honduras [30], was detected in 8 carriers of three families and was associated to 6 BC events. *BRCA2* c.6024dupG was found in 16 carriers of 5 apparently unrelated families. This mutation, although never recurrently, has been reported mostly in Latin American countries [31], which may suggest a founder effect in the south-west of Navarra. This duplication has showed the highest correlation with OC in our study population: 4 out of the 7 *BRCA2* positive OC identified in the HBOC study were associated to this mutation. In total, six mutation carriers developed ten different neoplasms, affecting ovary (4), breast (2), skin (1), endometrium (1), prostate (1) and lung (1). Three patients suffered multiple cancers. The information about the relationship between specific mutations in *BRCA1/2* and the clinical expression is limited; however, there is evidence that mutations in the central part of *BRCA2* (around exon 11), defined as the Ovarian Cancer Cluster Region, are associated with a higher OC risk and lower BC risk [32, 33]. Similarly, a Breast Cancer Cluster Region has been described at the end of *BRCA2* (c.7394-c.8904) [33]. Our data from mutations *BRCA2* c.6024dupG (exon 11) and c.7558C > T, with high association to OC and BC respectively, support these evidences.

Many studies have investigated the prognosis role of *BRCA* germ line mutations with inconsistent results. Some early studies suggested that *BRCA* associated BC and OC had a better prognosis than sporadic malignancies [34, 35], perhaps due to increased sensitivity to chemotherapy. *BRCA1/2* function as tumor suppressor genes and their proteins play an important role in repairing damaged DNA [16]. Deficiency of BRCA1/2 proteins results in carcinomas with a diminished capacity to repair DNA and, presumably, decreased ability to repair DNA breaks caused by chemotherapy. *BRCA1/2* mutation carriers seem to have an improved response to platinum-based chemotherapy [36]. However, whether the improved response translates into survival benefits still remains unclear.

More recent studies indicate that *BRCA* mutations do not have a protective effect [37]. In our population, women with BC and *BRCA* mutations had worse prognosis than those with sporadic cancer, diagnosed at the same age, with HR of 3.2 for *BRCA1* and 2.6 for *BRCA2*. Goodwin et al [38] suggested that prognosis was best predicted by the characteristic of the cancer developed by each individual rather than by their carrier status. Evaluating the effects of tumor histology and treatment-related variables was not the purpose of this study. However, we found that age at diagnosis, laterality (in *BRCA2* carriers), tumor stage and presence of multiple neoplasms were significantly associated with *BRCA* mutations. Interestingly, BC in HR-*BRCA*-negative and low-risk patients also presented at an earlier age than in the general population, but there were no significant differences with respect to the stage and laterality of the disease. On the other hand, their OS adjusted by age at diagnosis showed no differences with that of the general population, but it was significantly higher than in *BRCA1/2* carriers. These results may indicate that *BRCA* carriers present with more aggressive or severe tumor characteristics, and, consequently, a worse prognosis than sporadic BC. We cannot rule out, however, that it might be the result of an increased disease burden in *BRCA* positive patients, as they are also prone to suffer from other malignancies, in addition to BC.

In OC, diagnosis occurred 11–12 years earlier in all HBOC risk groups than in the general population, although only *BRCA1*-associated tumors were mostly bilateral and more advanced in tumor development. With respect to OS, sporadic OC seem to have a worse 5-year prognosis than *BRCA* associated carcinomas. Other studies also support the hypothesis of a protective OS effect of a *BRCA1/2* mutation carrier versus a non-carrier [39]. However, we observed that over a 15-year period, the apparent protective effect in *BRCA* is no longer present. Similar results have been described by other authors [40] suggesting that for *BRCA1/2* positive women with OC, the short-term survival advantage does not lead to a long-term OS better prognosis.

Finally, it is interesting to mention that, as it is well known, most high-risk families for HBOC do not carry a *BRCA* gene mutation [8]. However, they tend to share with them some of the phenotypic characteristics, such as, lower age at diagnosis and higher frequency of bilateral occurrence than cases in the low risk group and the general populations, which indicates that additional genetics and non-genetic factors remain to be identified.

This study has some strengths and limitations. It has an unbiased design with practically complete ascertainment of *BRCA* carriers in the studied population, as the Department of Medical Genetics is the reference center for genetic testing in Navarra. Additionally, the study sample comprises a large population-based cohort of BC and OC with reliable clinical and outcomes data on *BRCA* and non-*BRCA* carriers. However, the small number of *BRCA* families and cancer events in carriers may have limited the precision estimates of main effects and reduced statistical power of the study.

In summary, this retrospective study reviews HBOC due to mutations in *BRCA1/2* genes and describes the associated clinical and molecular features in Navarra. Additionally, it presents the state of the art of clinical practice for HBOC in our community, providing information on intermediate process and results indicators that will allow us to evaluate long-term efficiency of the cancer genetic testing program and its impact on public health.

## Conclusion

The spectrum of *BRCA* deleterious mutations in Navarra is similar to other Spanish regions, with some unique features. The possible founder effect of *BRCA2* c.6024dupG and its high association with OC must be taking into account when performing genetic testing. Overall, severe phenotypic features such as early diagnosis, high tumor grading, occurrence of multiple malignancies and bilateral location, are more frequent among *BRCA* carriers than in other cancer risk groups, conferring a poorer prognosis than those of sporadic origin. For BC, *BRCA* carriers have a worse OS rate at 5-, 10- and 15 years than cases from the general population. This study provides intermediate indicators that will help to further evaluate long-term prognosis of patients with HBOC and improve health care in our community.

## Supplementary information

**Additional file 1: Figure S1.** Inclusion criteria of the hereditary breast/ovarian cancer study.

**Additional file 2: Table S1.** BRCA1 mutations identified in this study. Germ line *BRCA1* pathogenic mutations, molecular change, frequencies, geographical origin of the families (NC = neighbouring communities; OSC = Other Spanish communities, OC = other communities) and associated tumors.

**Additional file 3: Table S2.** BRCA2 mutations identified in this study. Germ line *BRCA2* pathogenic mutations, molecular change, frequencies, geographical origin of the families (NC = neighbouring communities; OSC = Other Spanish communities, OC = other communities) and associated tumors.

## Data Availability

The datasets used and/or analysed during the current study are available from the corresponding author upon reasonable request.

## Abbreviations

BC: Breast Cancer
CDS: Coding DNA Sequence
CHN: Complejo Hospitalario de Navarra
CI: Confidence Interval
EC: Endometrium Cancer
HBOC: Hereditary Breast and Ovarian Cancer
HR: Hazard Ratio
KM: Kaplan-Meier
LC: Lung Cancer
OC: Ovarian Cancer
OR: Odds Ratio
OS: Overall Survival
PC: Pancreatic Cancer
PrC: Prostate Cancer
SC: Skin Cancer
SD: Standard Deviation
TNM: Tumor, Node and Metastases
