# Correction to: Genetic and clinical characterization of *BRCA*-associated hereditary breast and ovarian cancer in Navarra (Spain)

**DOI:** 10.1186/s12885-019-6458-7

**Published:** 2019-12-17

**Authors:** Ainara Ruiz de Sabando, Edurne Urrutia Lafuente, Fermín García-Amigot, Angel Alonso Sánchez, Lourdes Morales Garofalo, Sira Moreno, Eva Ardanaz, Maria A. Ramos-Arroyo

**Affiliations:** 1 Department of Medical Genetics, Complejo Hospitalario de Navarra (CHN), Pamplona, Spain; 2 Navarrabiomed, Pamplona, Spain; 3 IdiSNA, Navarra Institute for Health Research, Pamplona, Spain; 4 Navarra Public Health Institute, Pamplona, Spain; 5 CIBER Epidemiology and Public Health CIBERESP, Madrid, Spain

**Correction to: BMC Cancer (2019) 19:1145**

**https://doi.org/10.1186/s12885-019-6277-x**

Following publication of the original article [1], the authors reported an error in Fig. 2, where the color code of the text boxes is reversed. Figure 2**-amended** shows the correct color association between the text boxes and the different areas in the map: Navarra, neighbouring communities and other Spanish communities.

**Fig. 2:**
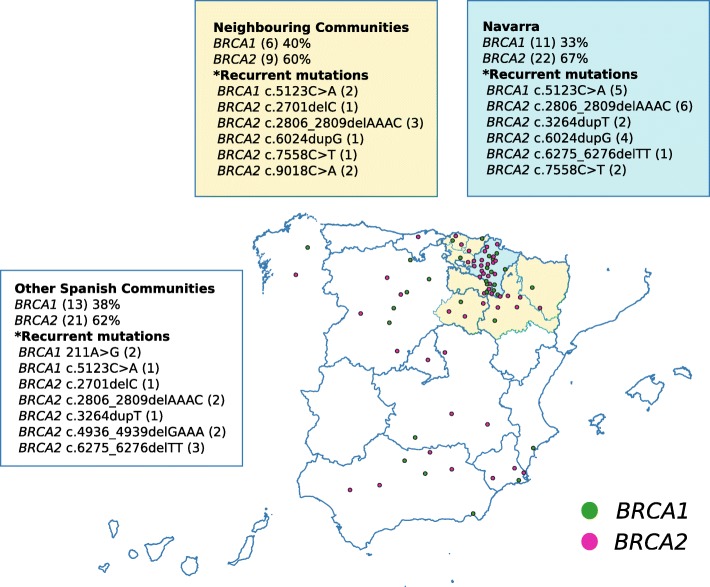
Geographical origin of the hereditary breast/ovarian cancer families in Navarra. (Map created with QGIS 3.0)

## References

[1] de Sabando R (2019). Genetic and clinical characterization of BRCA-associated hereditary breast and ovarian cancer in Navarra (Spain). BMC Cancer.

