# Supplementary material for: Genetic and clinical characterization of BRCA-associated hereditary breast and ovarian cancer in Navarra (Spain)
Source: BMC Cancer. 2019 Nov 27;19:1145. doi: 10.1186/s12885-019-6277-x (PMC6880350; doi:10.1186/s12885-019-6277-x)
Supplement: Supplementary file 1 — Additional file 1: Figure S1. Inclusion criteria of the hereditary breast/ovarian cancer study. [file 12885_2019_6277_MOESM1_ESM.doc]

Young age of diagnose of breast cancer (≤35 years).

Bilateral breast cancer +

Diagnose age <50, or

One first- or second-degree relative with breast cancer before the age of 60

Ovarian cancer (at any age) and

Breast cancer in the same patient, or

A relative with ovarian cancer, or

One first-degree relative with breast cancer before the age of 50, or

Two first- or second-degree relatives with average diagnose age ≤ 60

Breast cancer in the male +

One first-degree relative with breast cancer before the age of 50

Two first- or second-degree relatives with breast cancer before the age of 60.

Two or more first-degree relatives with breast cancer, and:

Average diagnose age ≤ 50 years, or

One of them bilateral.

Three first- or second-degree relatives with breast cancer (at least one must be a first degree relative) and,

Average diagnose age <60.
