# Supplementary material for: Genetic and clinical characterization of BRCA-associated hereditary breast and ovarian cancer in Navarra (Spain)
Source: BMC Cancer. 2019 Nov 27;19:1145. doi: 10.1186/s12885-019-6277-x (PMC6880350; doi:10.1186/s12885-019-6277-x)
Supplement: Supplementary file 2 — Additional file 2: Table S1. BRCA1 mutations identified in this study. Germ line BRCA1 pathogenic mutations, molecular change, frequencies, geographical origin of the families (NC = neighbouring communities; OSC = Other Spanish communities, OC = other communities) and associated tumors. [file 12885_2019_6277_MOESM2_ESM.doc]

| ***BRCA1***  **MUTATION CDS** | **MUTATION PROTEIN** | **OTHER NAMES** | **VARIANT TYPE** | **MOLECULAR CONSEQUENCE** | **PROTEIN CHANGE** | **No. FAMILIES / FREQ.** | | **ORIGIN** | **ASSOCIATED TUMORS CANCER REGISTRY** |
| --- | --- | --- | --- | --- | --- | --- | --- | --- | --- |
| c.68_69delAG | p.Glu23Valfs | 185delAG | Deletion | Frameshift variant | Stop 39 | 1 | 0,032 | OSC | - |
| c.131G>T | p.Cys44Phe | 250G>T | SNV | Missense variant | C44F | 1 | 0,032 | Navarra | Breast (1); Skin (1) |
| c.211A>G | p.Arg71Gly | R71G | SNV | Missense variant | R71G | 2 | 0,064 | OSC (2) | Ovary (2); Skin (1) |
| c.783T>G | p.Tyr261Ter | 902T>G | SNV | Nonsense | Y261* | 1 | 0,032 | OSC | Breast (2); Ovary (1) |
| c.798_799delTT | p.Ser267Lysfs | 916delTT | Deletion | Frameshift variant | Stop 285 | 1 | 0,032 | OC | Breast (1) |
| c.1386delG | p.Thr464Profs | 1505delG | Deletion | Frameshift variant | Stop 474 | 1 | 0,032 | Navarra | Ovary (2); Breast (1) |
| c.1961dupA | p.Tyr655Valfs | 2080insA | Duplication | Frameshift variant | Stop 672 | 1 | 0,032 | OSC | Ovary (2); Breast (1) |
| c.2900_2901dupCT | p.Pro968Leufs | - | Duplication | Frameshift variant | Stop 999 | 1 | 0,032 | Navarra | Breast (1) |
| c.3329_3330delAG | p.Lys1110Thrfs | 3448delAG | Deletion | Frameshift variant | Stop 1113 | 1 | 0,032 | Navarra | Breast (2); Skin (1) |
| c.3607C>T | p.Arg1203Ter | 3726C>T | SNV | Nonsense | R1203* | 1 | 0,032 | OSC | Breast (1) |
| c.3679C>T | p.Gln1227Ter | 3798C>T (Q1227X) | SNV | Nonsense | Q1227* | 1 | 0,032 | OSC | Breast (2) |
| c.3839_3843delCTCAGinsAGGC | p.Ser1280Terfs | 3958del5ins4 | Indel | Frameshift variant | Stop 1280 | 1 | 0,032 | OSC | Breast (3); Ovary (1) |
| c.4038_4039delAA | p.Gly1348Asnfs | - | Deletion | Frameshift variant | Stop 1354 | 1 | 0,032 | NC | Breast (1); Ovary (1) |
| c.4195_4196delAC | p.Thr1399Hisfs | 4314delAC | Deletion | Frameshift variant | Stop 1402 | 1 | 0,032 | NC | Breast (3) |
| c.4343delG | p.Ser1448Thrfs | - | Deletion | Frameshift variant | Stop 1455 | 1 | 0,032 | NC | Pancreas (1) |
| c.5054C>T | p.Thr1685Ile | 5173C>T | SNV | Missense variant | T1685I | 1 | 0,032 | NC | - |
| c.5123C>A | p.Ala1708Glu | 5242C>A | SNV | Missense variant | A1708E | 8 | 0,258 | Navarra (5);  NC (2); OSC (1) | Breast (4); Ovary (2); Pancreas (1) |
| c.5137G>T | p.Val1713Leu | - | SNV | Missense variant | V1713L | 1 | 0,032 | Navarra | Breast (2); Skin (1) |
| c.5144G>A | p.Ser1715Asn | - | SNV | Missense variant | S1715N | 1 | 0,032 | NC | Breast (1) |
| c.5152+5G>A | - | IVS18+5G>A | SNV | Intron variant | - | 1 | 0,032 | OSC | Ovary (2); Skin (2) |
| c.5194-2A>G | - | IVS19-2A>G | SNV | Splice acceptor variant | - | 1 | 0,032 | OSC | Breast (1) |
| Exon 1-13 deletion | - | - | LGR | - | - | 1 | 0,032 | OSC | Breast (1) |
| Exon 5-7 duplication | - | - | LGR | - | - | 1 | 0,032 | Navarra | Breast (2); Endometrium (1); Lung (1), Bone Marrow (2) |
| TOTAL  23 mutations |  |  |  |  | - | 31 | 1 | OSC (38.7%)  Navarra (35.5%)  NC (22.6%)  OC (3.2%) | 54 tumors |
