# Supplementary material for: Genetic and clinical characterization of BRCA-associated hereditary breast and ovarian cancer in Navarra (Spain)
Source: BMC Cancer. 2019 Nov 27;19:1145. doi: 10.1186/s12885-019-6277-x (PMC6880350; doi:10.1186/s12885-019-6277-x)
Supplement: Supplementary file 3 — Additional file 3: Table S2. BRCA2 mutations identified in this study. Germ line BRCA2 pathogenic mutations, molecular change, frequencies, geographical origin of the families (NC = neighbouring communities; OSC = Other Spanish communities, OC = other communities) and associated tumors. [file 12885_2019_6277_MOESM3_ESM.doc]

| ***BRCA2***  **MUTATION CDS** | **MUTATION PROTEIN** | **OTHER NAMES** | **VARIANT TYPE** | **MOLECULAR CONSEQUENCE** | **PROTEIN CHANGE** | **No. FAMILIES / FREQ.** | | **ORIGIN** | **ASSOCIATED TUMORS CANCER REGISTRY** |
| --- | --- | --- | --- | --- | --- | --- | --- | --- | --- |
| c.658_659delGT | p.Val220Ilefs | 886delGT | Deletion | Frameshift variant | Stop 223 | 1 | 0,019 | OSC | Breast (2); Endometrium (1) |
| c.1587delTinsCA | p.Glu532Argfs | 1815delTinsCA | Indel | Frameshift variant | Stop 534 | 1 | 0,019 | Navarra | Breast (1) |
| c.1929delG | p.Arg645Glufs | 2157delG | Deletion | Frameshift variant | Stop 659 | 1 | 0,019 | OSC | Breast (2) |
| c.2701delC | p.Ala902Leufs | 2929delC | Deletion | Frameshift variant | Stop 903 | 2 | 0,038 | NC (1); OSC (1) | Breast (3) |
| c.2806_2809delAAAC | p.Ala938Profs | 3034delAAAC | Deletion | Frameshift variant | Stop 959 | 11 | 0,208 | Navarra (6);  NC (3); OSC (2) | Breast (14); Colon (1); Thyroid gland (1), Bone Marrow (1) |
| c.3068delA | p.Asn1023Thrfs | 3296delA | Deletion | Frameshift variant | Stop 1042 | 1 | 0,019 | OSC | Breast (1) |
| c.3264dupT | p.Gln1089Serfs | 3492insT | Duplication | Frameshift variant | Stop 1098 | 3 | 0,057 | Navarra (2);  OSC (1) | Breast (2); Skin (1) |
| c.3744_3747delTGAG | p.Ser1248Argfs | 3972delTGAG | Deletion | Frameshift variant | Stop 1257 | 1 | 0,019 | NC | - |
| c.4132_4133delAC | p.Thr1378Serfs | - | Deletion | Frameshift variant | Stop 1380 | 1 | 0,019 | Navarra | Breast (1) |
| c.4677delT | p.Phe1559Leufs | - | Deletion | Frameshift variant | Stop 1567 | 1 | 0,019 | NC | - |
| c.4936_4939delGAAA | p.Glu1646Glnfs | 5164del4 | Deletion | Frameshift variant | Stop 1668 | 2 | 0,038 | OSC (2) | Breast (2); Ovary (1); Fossa Piriform (1) |
| c.5042_5043delTG | p.Val1681Glufs | 5270delTG | Deletion | Frameshift variant | Stop 1687 | 1 | 0,019 | Navarra | - |
| c.5200G>T | p.Glu1734Ter | - | SNV | Nonsense | E1734* | 1 | 0,019 | OSC | - |
| c.5216_5218insAAA | p.Thr1739Ter | - | Insertion | Nonsense | T1739* | 1 | 0,019 | NC | Breast (1); Skin (1) |
| c.5851_5854delAGTT | p.Ser1951Trpfs | 6076del4 | Deletion | Frameshift variant | Stop 1961 | 1 | 0,019 | OSC | Breast (1); Ovary (1) |
| c.6024dupG | p.Gln2009Alafs | 6252insG | Duplication | Frameshift variant | Stop 2017 | 5 | 0,094 | Navarra (4);  NC (1) | Ovary (4); Breast (2); Skin (1); Endometrium (1); Prostate gland (1); Lung (1) |
| c.6129delA | p.Gly2044Alafs | 6357delA | Deletion | Frameshift variant | Stop 2050 | 1 | 0,019 | OSC | Breast (1) |
| c.6275_6276delTT | p.Leu2092Profs | 6503delTT | Deletion | Frameshift variant | Stop 2098 | 4 | 0,076 | OSC (3);  Navarra (1) | Breast (4) |
| c.7558C>T | p.Arg2520Ter | 7786C>T | SNV | Nonsense | R2520* | 3 | 0,057 | Navarra (2);  NC (1) | Breast (6) |
| c.8167G>C | p.Asp2723His | 8395G>C | SNV | Missense variant | D2723H | 1 | 0,019 | NC | Breast (1) |
| c.8948_8953+5delATTCAGGTAAG | - | 9176del11 | Deletion | Intron variant | - | 1 | 0,019 | OC | Breast (1) |
| c.9018C>A | p.Tyr3006Ter | - | SNV | Nonsense | Y3006* | 2 | 0,038 | NC (2) | Breast (1) |
| c.9026_9030delATCAT | p.Tyr3009Serfs | 9254delATCAT | Deletion | Frameshift variant | Stop 3016 | 1 | 0,019 | OSC | - |
| c.9098_9099insA | p.Gln3034Serfs | 9326insA | Insertion | Frameshift variant | Stop 3042 | 1 | 0,019 | Navarra | Breast (2) |
| c.9117G>A | p.Pro3039= | 9345G>A | SNV | Synonymous variant | P3039P | 1 | 0,019 | Navarra | Breast (3); Esophagus (1) |
| c.9127G>T | p.Glu3043Ter | 9355G>T | SNV | Nonsense | Stop 3043 | 1 | 0,019 | Navarra | Breast (1); Prostate gland (1) |
| c.9253delA | p.Thr3085Glnfs | 9481delA | Deletion | Frameshift variant | Stop 3104 | 1 | 0,019 | Navarra | Ovary (1); Peritoneum (1) |
| c.9310_9311delAA | p.Lys3104Valfs | 9538delAA | Deletion | Frameshift variant | Stop 3109 | 1 | 0,019 | NC | Breast (1) |
| Exon 2 deletion | - | exon2del | LGR | - | - | 1 | 0,019 | OSC | Skin (1) |
| TOTAL  29 mutations |  |  |  |  |  | 53 | 1 | Navarra (41.5%)  OSC (32.1%)  NC (24.5%)  OC (1.9%) | 75 tumors |
